# Patient-reported outcome measures and surgery for Crohn’s disease: systematic review

**DOI:** 10.1093/bjsopen/zrad098

**Published:** 2023-10-26

**Authors:** Whei J Kim, Mohamad Iskandarani, Carlo A Manzo, Gianluca Pellino, Marc Martí Gallostra, Paris P Tekkis, Valerio Celentano, Christos Kontovounisios

**Affiliations:** Department of Surgery and Cancer, Imperial College London, London, UK; Department of Surgery and Cancer, Imperial College London, London, UK; Department of Colorectal Surgery, Chelsea and Westminster Hospital NHS Foundation Trust, London, UK; Colorectal Surgery, Vall d’Hebron University Hospital, Universitat Autonoma de Barcelona, UAB, Barcelona, Spain; Department of Advanced Medical and Surgical Sciences, Università degli Studi della Campania ‘Luigi Vanvitelli’, Naples, Italy; Colorectal Surgery, Vall d’Hebron University Hospital, Universitat Autonoma de Barcelona, UAB, Barcelona, Spain; Department of Surgery and Cancer, Imperial College London, London, UK; Department of Colorectal Surgery, Chelsea and Westminster Hospital NHS Foundation Trust, London, UK; Department of Colorectal Surgery, Royal Marsden NHS Foundation Trust, London, UK; Department of Surgery and Cancer, Imperial College London, London, UK; Department of Colorectal Surgery, Chelsea and Westminster Hospital NHS Foundation Trust, London, UK; Department of Surgery and Cancer, Imperial College London, London, UK; Department of Colorectal Surgery, Chelsea and Westminster Hospital NHS Foundation Trust, London, UK; Department of Colorectal Surgery, Royal Marsden NHS Foundation Trust, London, UK

## Abstract

**Background/Aims:**

Crohn’s disease is an inflammatory bowel disease with up to 50 per cent of patients requiring surgery within 10 years of diagnosis. Patient-reported outcome measures (PROMs) are vital to monitor and assess patient health-related quality of life (HRQoL). This systematic review aims to evaluate PROMs within studies for perioperative Crohn's disease patients.

**Methods:**

Articles from MEDLINE, Embase, Emcare and CINAHL databases were searched to find studies relating to the assessment of HRQoL in perioperative Crohn's disease patients using PROMs and patient-reported experience measures (PREMs) from 1st January 2015 to 22nd October 2023. Bias was assessed using the ROBINS-I tool was used for non-randomized interventional studies and the Cochrane RoB2 tool was used for randomized trials.

**Results:**

1714 journal articles were filtered down to eight studies. Six studies focused on ileocaecal resection, one on perianal fistulas and one on the effects of cholecystectomy on patients with Crohn's disease. Within these articles, ten different PROM tools were identified (8 measures of HRQoL and 2 measures of functional outcome). Overall improvements in patient HRQoL pre- to postoperative for ileocaecal Crohn's disease were found in both paediatric and adult patients. Outcomes were comparable in patients in remission, with or without stoma, but were worse in patients with a stoma and active disease.

**Conclusion:**

There are significant variations in how PROMs are used to evaluate perioperative Crohn's disease outcomes and a need for consensus on how tools are used. Routine assessments using an internationally accepted online platform can be used to monitor patients and support areas of treatment pathways that require further support to ensure high standards of care. They also enable future statistical comparisons in quantitative reviews and meta-analyses.

## Teaser

Crohn’s disease (CD) is an inflammatory bowel disease that often requires surgical intervention, making patient-reported outcome measures (PROMs) an important tool for monitoring and assessing patient health-related quality of life (HRQoL). This systematic review evaluates the use of PROMs within surgical CD patients and is able to identify eight studies which collectively utilize eight measures of HRQoL and two measures of function. This review highlights the need for a consensus on how these tools are used and to monitor patients, support treatment pathways and enable future quantitative reviews and meta-analyses.

## Introduction

Crohn’s disease (CD) is a chronic inflammatory bowel disease (IBD) affecting the entirety of the gastrointestinal tract, but most commonly within the distal ileum of the small intestine^1^. CD can manifest with a variety of presentations at multiple sites and surgical management is complex. CD has a high incidence of perianal disease with a 20-year cumulative incidence of up to 42 per cent^2^. Perianal fistulas are the most common presentation of perianal disease and often require surgical intervention, with a significant proportion of perianal CD patients eventually requiring a permanent stoma^3,4^. Despite the medical therapies available for the management of symptoms, surgical intervention remains a mainstay for the treatment pathway of CD, with approximately 50 per cent of patients requiring surgery within 10 years of diagnosis, with historical reports of surgical recurrence being as high as 80 per cent in previously operated patients^1,5^. Surgery cannot cure CD, but is indicated for complications of the disease and to improve patients’ quality of life. Despite the high risk of surgery associated with the disease, the regular assessment of patient-reported outcome measures (PROMs) to assess patient health-related quality of life (HRQoL) is lacking^6,7^. A multicentre study assessing national perioperative assessments for surgically managed CD patients in Italy found that PROMs and functional outcomes were being assessed in only 1 per cent of patients following surgery^8^.

PROMs are crucial to monitor the impact of surgical interventions in CD. These PROMS are becoming increasingly established, not only during perioperative management but also during remission and medical management. The Food and Drug Administration has emphasized the need for the use of patient-reported outcomes as primary outcomes within clinical trials^9^. This study aims to investigate and systematically evaluate the use of PROMs within studies across perioperatively managed CD patients.

## Methods

### Search strategy and article selection

A comprehensive systematic literature search was performed through the MEDLINE, Embase, Emcare and CINAHL (the Cumulative Index to Nursing and Allied Health Literature) databases using the Healthcare Databases Advanced Search (HDAS) platform in order to identify relevant articles investigating the use of PROMs, patient-reported experience measures (PREMs) or other HRQoL tools in CD patients following surgical intervention.

### Data of interest

The search included all English journal articles from 1st January 2015 to the date of the search (23rd October 2023). The main search terms used were: ‘Crohn’s Disease’, ‘Granulomatous Enteritis’, ‘Crohn’s Colitis’, ‘Regional Ileitis’, ‘Terminal Ileitis’, ‘Ileocolitis’, alongside ‘Surg*’, ‘Surg* Manag*’, ‘Operat*’, ‘Colectomy’, ‘Bowel Resection’, and ‘Patient-Reported Outcome’, Patient-Reported Experience’, ‘Patient Experience’, ‘PRO’, ‘PROM’, ‘PREM’.

### Inclusion and exclusion

Articles containing PROMs data from surgically managed patients with Crohn’s disease were included, meaning that data encompassing all forms of IBD (CD, ulcerative colitis and indeterminate IBD) that did not distinguish CD patients were excluded. Similarly, results that included both surgical and non-surgical PROMs data that did not separate surgical data only were also excluded.

Additional searches were also done into the bibliographies of included studies to identify further articles.

Articles were independently screened by two authors (W.J.K. and M.I.), and at full-text screening, disagreements were discussed, and a collective decision was made whether to include or exclude.

### Data extraction

Articles were subdivided into separate manifestations of Crohn’s disease (for example, ileal/ileocaecal, perianal) and the outcome measures used within these studies were analysed to compare how they are being used, as well as their associated benefits and limitations.

Quantitative PROMs outcome data were extracted; however, as PROMs tools within CD surgery are still in their infancy, meaningful statistical analysis was not possible. This is due to differences between what these outcome measures assess; accumulation of data sets and comparisons were difficult to achieve.

### Bias and quality of study assessment

The ROBINS-I tool was used for non-randomized interventional studies and the Cochrane RoB2 tool was used for randomized trials^10,11^, the results of which are summarized in *Table S1a & S1b*.

This review was completed following the PRISMA guidelines^12^ (*Table S2*) and the review has been registered on PROSPERO (registration identifier: CRD42022349869). A separate review protocol was not prepared.

## Results

### Search results

**Fig. 1:**
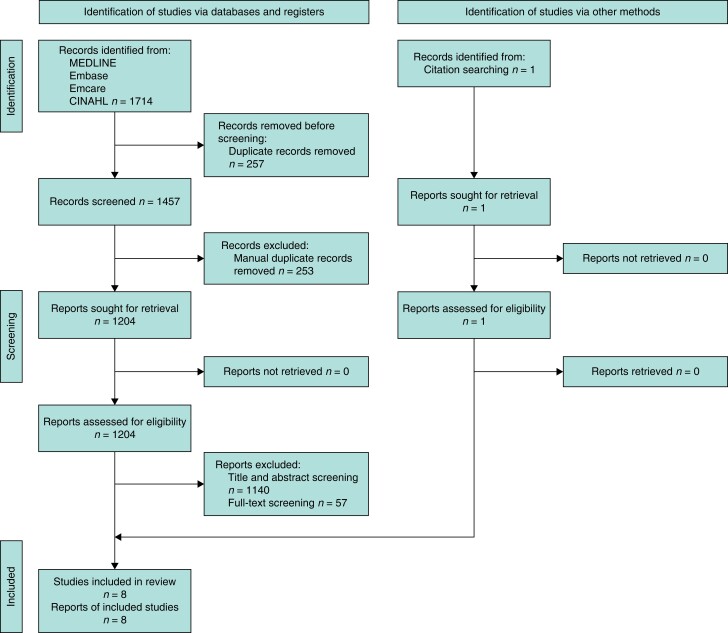
PRISMA 2020 flowchart demonstrating the study selection process

A total of 1714 articles were identified and were subjected to automated deduplication (using HDAS and Zotero’s deduplication processes) followed by manual deduplication, which removed a total of 510 articles. A final 1204 articles remained for screening, and following title and abstract screening, 1140 articles were excluded, leaving 64 articles for full-text screening; of these, 57 articles were excluded as they lacked PROMs within colorectal surgery, PROMs data were not exclusive to CD (namely, data were grouped with all other IBD patients), PROMs data were not presented within the paper, or patients were not treated surgically. Seven articles^13–19^ were found to contain relevant information and a single further article^20^ was found through screening bibliographies of articles in the database, leaving eight articles^13–20^ focusing solely on CD patients following surgery for evaluation for this review (*Fig. 1*). Summaries of the studies, the key findings and information about the PROMs included are represented in the tables below (*Tables 1*, *2* and *3*).

**Table 1:** Summary table of study methods included in analysis

Reference	Country	Study design	PROM/instrument	*n*	Patients included
Diederen *et al*.^13^	Netherlands	Cross-sectional study	SF-36 COREFO Patient Satisfaction Scale	80	Primary ileocaecal resection for CD during childhood (< 18 years)
Dipasquale *et al*.^14^	Italy	Cross-sectional study	Modified IMPACT III	10	Laparoscopic-assisted ileocaecal resection for CD during childhood (≤ 18 years)
Gerdin *et al*.^15^	Sweden	RCT	SF-36 VAS	36	CD patients allocated to receiving either medical treatment (thiopurines) or surgical intervention (ileal or ileocaecal resection)
Ponsioen *et al*.^16^	Netherlands and UK	RCT	SF-36 IBDQ	143	Patients with active CD of the terminal ileum were randomly allocated to either surgical intervention (ileocaecal resection) or medical intervention (infliximab)
Abdalla *et al*.^17^	USA	Cross-sectional study	MIBDI SIBDQ PROMIS	4733	CD patients who reported either having or not having a stoma within 6 or more months
Adegbola *et al*.^18^	UK	Prospective cohort study	MYMOP2 Decisional Regret Scale	21	CD fistula patients undergoing video-assisted anal fistula treatment
Koutroumpakis *et al*.^19^	USA	Prospective cohort study	SIBDQ	834	CD patients who either had or had not undergone cholecystectomy
Wright *et al*.^20^	Australia and New Zealand	RCT	SF-36 IBDQ	174	CD patients allocated to either having 6-month postoperative colonoscopy (active care) or no colonoscopy (standard care) following surgical bowel resection

CD, Crohn's disease; PROM, patient-recorded outcome measure; SF-36, Short Form Health Survey 36; COREFO, colorectal functional outcome; VAS, visual analogue scale; IBDQ, inflammatory bowel disease questionnaire; MIBDI, Manitoba IBD Index; SIBDQ, short inflammatory bowel disease questionnaire; PROMIS, Patient-Reported Outcomes Measurement Information System; MYMOP2, Measure Your Medical Outcome Profile 2.

**Table 2:** Summary table of key findings across the studies included in analysis

Reference	Key findings
Diederen *et al*.^13^	Surgically treated patients showed significantly worse physical-related scores when compared to general populations: mean(s.d.); CD 46.9(10.0) *versus* general 54.3(6.5), *P* < 0.001. Mental-related health scores were not found to be significantly different: mean(s.d.); CD 48.5(10.5) *versus* general 49.9(9.4), *P* = 0.779. Patients with active disease demonstrated significantly lower physical health scores: mean(s.d.); general 54.3(6.5) *versus* active CD 36.8(6.2), *P* < 0.001. Patients with remissive disease demonstrated significantly lower physical health scores: mean(s.d.); general 54.3(6.5) *versus* remissive CD 51.5(7.8) *P* = 0.027. Only the patients with active disease demonstrated significantly worse mental health outcomes when compared to the general population: mean(s.d.); general 49.9(9.4) *versus* active CD 41.6(12.6), *P* < 0.001.
Dipasquale *et al*.^14^	Median IMPACT III scores from 12 and 6 months before surgery of 43.2 and 34.5, respectively, improved significantly to 65.4 (*P* < 0.001) across all dimensions of the questionnaire. Patient symptoms such as blood in the stool, abdominal pain and fatigue had significantly reduced from 6 months before to 12 months after surgery (*P* < 0.001). Patients reported having a significant increase in appetite, weight, and a better ability to perform activities of daily living (*P* < 0.001). Stool frequency also decreased; however, this difference was not significant (*P* = 0.223).
Gerdin *et al*.^15^	Surgically treated patients at 1 year demonstrated significantly better social function SF-36 scores (median(range); 9 (5–10) *versus* 10(8–10) in the medical treatment group, *P* = 0.010). These differences became comparable at 3 and 5 years. General health scores were better in the surgical treatment group, but this difference was not significant (median(range); 15(10–22) *versus* 10(10–13) in the medical treatment group, *P* = 0.060). Vitality scores were worse in the surgically treated group (median(range); 16(9–18) *versus* 16(10–18) in the medically treated group, *P* = 0.060), but this was not significant. VAS scores were comparable at all points throughout the trial.
Ponsioen *et al*.^16^	Surgically treated patients demonstrated significantly worse mean IBDQ scores at 2 weeks (140.1 *versus* 160.8 in the medical treatment group, *P* < 0.001). These differences became comparable from 6 weeks to 12 months. Surgically treated patients demonstrated significantly worse mean overall SF-36 scores at 2 weeks compared to medical treatment (*P* < 0.001); however, at 6 and 9 months, the surgical treatment group had significantly better scores (*P* = 0.017 and *P* = 0.010, respectively). When dividing the SF-36 into its physical and mental component scores, the surgical treatment group demonstrated significantly worse mean physical component scores at 2 weeks (*P* < 0.001); however, these became significantly better than the medical treatment group at 6, 9 and 12 months (*P* = 0.011, *P* = 0.004, and *P* = 0.040, respectively). Mean mental component scores of the SF-36 were comparable throughout the follow-up period.
Abdalla *et al*.^17^	Patients with active disease demonstrated comparable mean SIBDQ scores between patients with or without a stoma: mean(s.d.); 4.5(1.1) *versus* 4.1(1.1) (no stoma *versus* stoma, respectively), *P* > 0.050. Patients with remissive disease demonstrated comparable mean SIBDQ scores between patients with or without a stoma: mean(s.d.); 5.8(0.8) *versus* 5.8(0.8) (no stoma *versus* stoma, respectively), *P* > 0.050. Patients with a stoma in active disease demonstrated significantly worse PROMIS scores in the domains: fatigue, pain interference and social satisfaction than those with no stoma. PROMIS scores in patients with remissive disease were comparable in all domains.
Adegbola *et al*.^18^	Median MYMOP2 scores relating to symptoms of pain decreased significantly following surgery: median(range); preoperative: 4(1–6) *versus* postoperative: 1(0–4), *P* < 0.001. Median MYMOP 2 scores relating to symptoms of discharge decreased significantly following surgery: median(range); preoperative: 4(1–6) *versus* postoperative: 1(0–5), *P* < 0.001. 81% of patients agreed or strongly agreed that they had made the correct decision about their surgery. 71% of patients agreed or strongly agreed that they would undergo their procedure again. 95% of patients disagreed or strongly disagreed that the choice they made did them harm.
Koutroumpakis *et al*.^19^	Upon univariate analysis, patients who had undergone cholecystectomy were associated with lower mean SIBDQ scores: mean(s.d.); 42.6(11.2) *versus* 49.8(11.7), with and without cholecystectomy, respectively; OR = 2.24, *P* < 0.001. Upon multivariate analysis, after controlling for potential confounders, such as age, gender, smoking status and BMI, those with cholecystectomy were found to be associated with worse SIBDQ scores (adjusted OR = 0.38, *P* < 0.001). Upon univariate analysis within a subgroup of patients with ileal CD, mean SIBDQ scores were lower in those who had undergone cholecystectomy (mean(s.d.); 42(11) *versus* 50(11), with and without cholecystectomy, respectively, *P* < 0.001). Upon multivariate analysis within the same subgroup, after controlling for confounders, scores were significantly lower in those who had undergone cholecystectomy (adjusted OR = 0.47, *P* = 0.002).
Wright *et al*.^20^	Mean physical component scores of the SF-36 showed significant improvements from pre- to postoperatively: preoperative PCS mean(s.d.): 40(21) *versus* postoperative PCS means(s.d.): 68(21), 71(20), 72(21) (at 6, 12 and 18 months, respectively), *P* < 0.001. Mean mental component scores of the SF-36 showed significant improvement from before to after surgery: preoperative MCS mean(s.d.): 44(22) *versus* postoperative MCS means(s.d.): 68(20), 70(19), 70(21) (at 6, 12 and 18 months, respectively), *P* < 0.001. Mean IBDQ scores demonstrated a significant improvement from pre- to postoperatively: preoperative mean(s.d.): 125 [35) *versus* postoperative means(s.d.): 171(33), 175(31), 175(34) (at 6, 12 and 18 months, respectively), *P* < 0.001.

CD, Crohn's disease; VAS, visual analogue scale; SF-36, Short Form Health Survey 36; PCS, physical component score; MCS, mental component score; IBDQ, inflammatory bowel disease questionnaire; SIBDQ, short inflammatory bowel disease questionnaire; PROMIS, Patient-Reported Outcomes Measurement System; MYMOP2, Measure Your Medical Outcome Profile 2.

**Table 3:** Summary table of the patient-reported outcome measures used within studies

PROM	CD/IBD-specific	Tool validation	Components
SF-36^21^	No	Yes, although validation has not been tested for CD	A 36-item questionnaire consisting of 8 scales summarized into 2 main components: Physical health component (physical functioning, role—physical, bodily pain, and general health scales) Mental health component (vitality, social functioning, role—emotional, and mental health scales) Range: 0–100 A higher score indicates better outcomes
COREFO^22^	No	Yes	Colorectal functional outcome measure consisting of 27 questions assessing 5 domains (incontinence, social impact, frequency, stool-related aspects, and need for stool-related medication) Range: 0–100 A lower score indicates better outcomes
IMPACT III^23^	Yes	Yes	A measure of HRQoL in paediatric IBD patients consisting of 35 questions of functional outcomes and HRQoL grouped into 5 domains (systemic and IBD symptoms, body image, energy, embarrassment, and worries or concerns about IBD) This was modified by Dipasquale *et al*. for ease over phone interviews (15 questions in 4 domains) Range: 15–75 A higher score indicates better outcomes
VAS^24^	No	Yes, although validation has not been tested for CD	Pain rating scale measured by marking on a continuous 10 cm line that represents a scale from ‘no pain’ to ‘worst pain’ Scores are recorded by measuring the distance in millimetres Range: 0–100 A lower score indicates better outcomes
IBDQ^25^	Yes	Yes	A 32-question scale divided into four domains (bowel symptoms, systemic symptoms, emotional function, social function) scored using a 7-point Likert scale from ‘worst health’ (1 point) to ‘best health’ (7 points) Range: 32–224 A higher score indicates better outcomes
MIBDI^26^	Yes	Yes	A single-item disease activity measure using a 6-point scale that best describes the patient’s disease activity in the past 6 months, ranging from ‘constantly active, giving me symptoms every day’ to ‘I was well in the past 6 months’ Range: 1–6 A higher score indicates better outcomes (scores of 5 or 6 are defined as clinical remission)
SIBDQ^27^	Yes	Yes	A shortened version of the original IBDQ consisting of 10 items divided into 4 domains (bowel symptoms, systemic symptoms, emotional function, social function) scored using a 7-point Likert scale from ‘worst health’ (1 point) to ‘best health’ (7 points) Range: 10–70 Higher score indicates better outcomes
PROMIS^28^	No	Yes	An adaptive online tool used to measure the domains: anxiety, depression, fatigue, sleep disturbances, social satisfaction, sexual interest and satisfaction and pain interference in the study by Abdalla *et al*. Scores are calibrated using a *t*-score metric to produce a mean for the domain A higher score indicates higher level of the measured domain (for example, higher level of anxiety; higher level of social satisfaction)
MYMOP2^29^	No	Yes	Patients were asked to rate their 2 most important symptoms using a 6-point Likert scale by Adegbola *et al*. Range: 1–6 for each symptom A lower score indicates better outcomes
Decisional Regret Scale^30^	No	Yes	Scale to measure a patient’s regret regarding their treatment decision using a 5-point Likert scale Range: 1 (strongly agree)—5 (strongly disagree)

PROM, patient-reported outcomes measure; CD, Crohn's disease; IBD, inflammatory bowel disease; SF-36, Short Form Health Survey 36; COREFO, colorectal functional outcome; VAS, visual analogue scale; IBDQ, inflammatory bowel disease questionnaire; MIBDI, Manitoba IBD Index; SIBDQ, short inflammatory bowel disease questionnaire; PROMIS, Patient-Reported Outcomes Measurement Information System; MYMOP2 = Measure Your Medical Outcome Profile 2.

### Ileal/ileocaecal disease

Diederen *et al*.^13^ and Dipasquale *et al*.^14^ were two studies that assessed the outcomes of paediatric patients following ileocaecal resection. Diederen *et al*.^13^ assessed the outcomes of 80 patients who had undergone primary ileocaecal resection during childhood (defined by age < 18 years); however, quality of life was only assessed in patients who had reached adulthood at the time of the study (66/80 patients, median follow-up 102.9 months (i.q.r. 46.5–190.0)) as there was no single validated, standardized tool for assessing HRQoL in both adult and paediatric patients. The study used the Short Form Health Survey 36 (SF-36) to assess HRQoL, the results of which were compared to mean scores of the general national population. They utilized the patient-reported colorectal function outcome (COREFO) questionnaire to assess colorectal function, which was compared to a separate cohort of patients with normal colorectal function following cholecystectomy; they also assessed the patients’ satisfaction with the surgical procedure^13^.

Dipasquale *et al*.^14^ measured pre- and postoperative HRQoL using a modified IMPACT III questionnaire, an IBD-specific questionnaire for paediatric patients. Scores were obtained 12 and 6 months before surgery and were compared to the 12-month postoperative results of the same patients. Results between 6 months before surgery and 12 months after surgery were compared and statistically analysed^14^.

Overall, Dipasquale *et al*.^14^ were able to find that median IMPACT III scores from 12 and 6 months preoperatively showed significant improvement across all dimensions of the questionnaire^14^. When comparing HRQoL outcomes against general populations, Diederen *et al*.^13^ found an overall conclusion that paediatric primary ileocaecal resection patients had reported a significantly worse physical-related quality of life. However, mental-related health scores were comparable^13^.

Diederen *et al*.^13^ also performed subgroup analysis, dividing the cohort into active disease and remission, and compared scores against the general population. Physical health scores were significantly worse in both active disease and remission against the population, while mental health scores were only significantly worse in patients with active disease; scores were comparable in remissive patients compared to the general population^13^.

Within patients with active disease, COREFO scores were worse in all domains (incontinence, social impact, defecation frequency, stool-related aspects, and use of medication to thicken bowel movements) compared to a cohort of normal colorectal function. Patients with clinical remission were found to be only significantly worse in social impact domain^13^.

Dipasquale *et al*.^14^ found that patient symptoms such as blood in the stool, abdominal pain and fatigue had significantly improved from 6 months before, 12 months after surgery. Patients also reported having significant improvements in appetite and weight and a better ability to perform activities of daily living^14^.

School absences were reported in almost all patients before surgery, caused by abdominal pain, fatigue and diarrhoea. However, at 1 year following surgery, school absence reduced significantly. Social functioning scores, consisting of a patient’s ability to play, go out, play sport, and travel, significantly improved after surgery^14^.

Both studies found high levels of satisfaction following surgery^13,14^. Dipasquale *et al*.^14^ found that emotional functioning results demonstrated a significant reduction of feelings of anger, injustice and embarrassment due to Crohn’s at 12 months and receiving surgery did not affect the patients’ concerns for future health problems (*P* = 0.202). Diederen *et al*.^13^ found that the number of patients who rated their satisfaction following surgery as ‘very satisfied’ was the greatest at 42/80. A further 23 were ‘satisfied’, nine were ‘neutral’, four were ‘dissatisfied’ and two were ‘very dissatisfied’. Lower satisfaction was found to be correlated to a higher level of disease activity^13^. Dipasquale *et al*.^14^ also reported that almost all patients were completely satisfied with the surgery outcomes.

Three studies^15–17^ assessed HRQoL in CD patients receiving different treatments. Gerdin *et al*.^15^ and Ponsioen *et al*.^16^ conducted RCTs comparing medical treatment *versus* surgical resection. Gerdin *et al*.^15^ investigated HRQoL using the SF-36 and the visual analogue scale (VAS), and Ponsioen *et al*.^16^ utilized the inflammatory bowel disease questionnaire (IBDQ) as well as the SF-36.

Abdalla *et al*.^17^ compared HRQoL between CD patients with or without a stoma using a shortened version of the IBDQ (SIBDQ) and the Patient-Reported Outcomes Measurement Information System (PROMIS) as well as patient-reported functional outcomes using the Manitoba IBD Index (MIBDI). They recruited patients from a large online cohort of adult patients with IBD and subcategorized them, using MIBDI results, by disease status (remission or active disease) to avoid any confounding caused by this factor^17^.

Ponsioen *et al*.^16^ found that the surgical treatment group had significantly worse mean IBDQ scores at 2 weeks; however, these scores subsequently improved and became comparable from 6 weeks. Mean IBDQ scores of the surgical treatment group were higher than the medical treatment group at 6, 9 and 12 months; however, these differences were not significant.

Similar trends were observed in the mean combined SF-36 scores. The surgical treatment group had significantly lower scores than the medical treatment group at 2 weeks, but these became comparable at 6 weeks. At 6 and 9 months, the surgical treatment group demonstrated significantly better mean combined SF-36 scores, although this significance was lost at 12 months^16^.

When dividing the SF-36 into its physical and mental component scores, physical component scores in the surgical treatment group were significantly worse at 2 weeks but significantly better at 6, 9 and 12 months compared to the medical treatment group. Mental component scores in the surgical treatment group were worse than the medical treatment group at 2 weeks, 6 weeks and 3 months. At 6, 9 and 12 months, the surgical treatment group demonstrated higher mean mental component scores; however, none of these differences were statistically significant^16^.

Gerdin *et al*.^15^ found that at 1-year follow up, the surgically treated cohort demonstrated significantly better social function SF-36 scores. This difference, however, was no longer observed at 3 and 5 years. While better general health scores were also found in the surgical treatment group, this difference was not significant. Vitality scores were worse in the surgically treated group, but again, this was not statistically significant. Additionally, VAS scores showed no statistical differences^15^.

Abdalla *et al*.^17^ found that the mean SIBDQ scores between patients with or without a stoma for both active disease and remission were not significantly different. For those in clinical remission, in all six domains of the PROMIS tool (anxiety, depression, fatigue, pain interference, sleep disturbances and social satisfaction), differences were found to be insignificant. However, in those with active disease, PROMIS scores in the domains fatigue, pain interference, and social satisfaction were significantly worse in those with a stoma than those without.

In this study^17^, as well as assessing and comparing the HRQoL of CD patients with or without a stoma, Abdalla *et al*.^17^ also compared outcomes of sexual interest and satisfaction in those with or without a stoma using PROMIS. Results were stratified by gender and, overall, there were no statistical differences found between those with or without a stoma in all domains for all genders^17^.

Wright *et al*.^20^ investigated the effects of bowel resection on the HRQoL of CD patients following their RCT^31^ comparing the outcomes of patients receiving a standard care arm *versus* an active care arm (consisting of medical therapy plus a 6-month postoperative colonoscopy). In order to assess patients’ HRQoL, the researchers used the SF-36 as well as the disease-specific IBDQ. Measures of HRQoL were recorded before surgery, as well as at 6, 12 and 18 months after surgery^20^.

The SF-36 results were divided into a physical component score and a mental component score. Results demonstrated a significant improvement of both the mean physical and mental scores from before to after surgery ^20^.

Mean IBDQ scores also demonstrated similar trends, showing a significant pre- to postoperative improvement in HRQoL throughout the follow-up period^20^.

### Perianal disease

The study by Adegbola *et al*.^18^ assessed the PROMs results of CD patients with perianal disease. They investigated the HRQoL outcomes of patients with perianal fistulas following video-assisted anal fistula treatment (VAAFT) using the Measure Your Medical Outcome Profile 2 (MYMOP2) quality-of-life questionnaire as a primary endpoint at 6 weeks following surgery. These results were compared to the patients’ results 6 weeks before surgery, and they also assessed patients’ attitudes and feelings of regret using the Decisional Regret Scale^18^.

Median MYMOP2 scores relating to symptoms of pain and discharge demonstrated significant improvements from 6 weeks before to 6 weeks after surgery^18^.

The results also demonstrated overall positive sentiments towards the procedure, with 81 per cent of patients agreeing or strongly agreeing that they had made the correct decision, as well as 71 per cent of patients agreeing or strongly agreeing that they would undergo the procedure again. Furthermore, 95 per cent of patients disagreed or strongly disagreed that the choice they made did them harm^18^.

### Abdominal surgery secondary to Crohn’s disease

CD patients have been found to be associated with a decreased ability to absorb bile acids due to CD-related tissue dysfunction or bowel resection, leading to a higher incidence of gallstones, and this is linked to increased rates of cholecystectomy^19,32^. Koutroumpakis *et al*.^19^ sought to investigate the effects of cholecystectomy on the HRQoL of CD patients using the SIBDQ between patients with (151 patients) or without (683 patients) cholecystectomy.

Koutroumpakis *et al*.^19^ were able to demonstrate that those who underwent cholecystectomy had a significantly worse quality of life compared to those with CD who did not require cholecystectomy^19^. The cholecystectomy group was associated with significantly lower mean SIBDQ scores upon univariate analysis and after controlling for potential confounders, such as age, gender, smoking status and BMI. Multivariable analysis demonstrated that patients who had a cholecystectomy were associated with significantly worse SIBDQ scores^19^.

Subgroup analysis was performed on patients with ileal CD to assess the impact of cholecystectomy on the development of dysplasia because they would have reduced absorption of bile, leading to increased secretion of bile and thus increased spilling into the colon. Within this subgroup, patients who had undergone cholecystectomy were found to have significantly worse SIBDQ scores with uni- and multivariable analysis^19^.

## Discussion

This review demonstrated significant heterogeneity in the PROMs tools utilized to assess patient outcomes following CD surgery. Of the ten tools identified, eight are used to assess HRQoL, with the remaining two being used to assess patient-reported functional outcomes. Clearly, there are a wide variety of tools available to assess HRQoL within perioperative CD patients, and this study was able to identify areas within the studies included in this review where improvements could be made.

The main limitation found within the study by Diederen *et al*.^13^ was that patient HRQoL scores were compared to the mean values of the general population of adults aged 26–35. A better alternative could have been to compare pre- and postoperative HRQoL if that information had been available, highlighting the need for PROMs-focused prospective studies in CD, which would allow for a clearer representation of the effects of the surgical intervention. The median follow-up time for HRQoL assessment within this cohort was 102.9 months (i.q.r. 46.5–190.0). Due to the extended follow-up period, it may be difficult to draw meaningful conclusions about the direct effects of surgical intervention during childhood on a patient’s HRQoL. While ileocaecal resection during childhood can have lifelong effects, data are lacking regarding subsequent interventions and management steps implemented up until the point of follow-up, which could act as confounding factors. The effects of childhood ileocaecal resection at the time of follow-up on this cohort may be less representative of the effects of the intervention alone.

Within this study^13^, patients could have had a significant pre- to postoperative improvement; however, by comparing to a baseline of a general population, these trends would be masked. More frequent/routine recording of PROMs would allow for more representative observations of how the disease progression and various interventions of CD could affect HRQoL over its course.

This limitation was not present within the study by Dipasquale *et al*.^14^ as, despite being a retrospective analysis, the data were present due to routine records of patient scores within the centre. Through regular close monitoring of PROMs, clinical teams would be able to spot patients that were not coping well and provide further medical, emotional and social support, using the data to potentially signpost patients for further care.

Wright *et al*.^20^ noted the improvement found at 6 months following surgery was maintained at 12 and 18 months. This study was able to demonstrate the sustained improvement in HRQoL following surgical intervention. The conclusion that bowel resection is able to produce a long-term improvement of HRQoL within this trial is significant due to the lack of PROMs data at longer follow-up periods.

Comparing this study^20^ (which demonstrated an improvement from pre- to postoperative outcomes) to the study by Gerdin *et al*.^15^, they were also able to find significantly better outcomes within surgically treated patients when compared to those who were treated with medical therapy at 1- year of follow-up. This benefit, however, was seemingly lost at 3 and 5 years of follow-up.

The two studies^15,20^ highlight the importance of long-term monitoring of patients’ HRQoL through the use of PROMs in CD patients following surgery. CD is a lifelong disease, and the effects of surgery are lifelong too. Routine assessment using PROMs would allow for the analysis of HRQoL outcomes at even longer follow-up periods, allowing researchers to continuously break the ceiling of the definition of the best standard of care, while allowing clinicians and patients to make informed decisions based on long-term empirical data.

Regular PROMs assessments, both short- and long-term, are essential for understanding a patient’s HRQoL. In the study by Ponsioen *et al*.^16^, frequent PROMs assessments were performed, from 2 weeks after baseline up to 12 months of follow-up. Trends of how HRQoL outcomes change over time and differ from medical treatment groups were observed. The study by Gerdin *et al*.^15^, which also compares two randomized arms of surgical and medical intervention, was designed so that follow-up would be performed at 1, 3 and 5 years. Although Gerdin *et al*.^15^ show significantly better social functioning SF-36 scores for the surgical group, as well as trends to higher general health scores at 1 year, this initial follow-up interval is relatively long. While assessing long-term outcomes is important for such a lifelong disease, it is also important to assess patient outcomes in the short term. The results from Ponsioen *et al*.^16^ are able to demonstrate how outcomes may change within that first year after intervention, and that in fact mean IBDQ and SF-36 scores in the surgical treatment group are initially significantly worse, and become comparable over time, becoming significantly better than the medical treatment group at some points. Together, these studies^15,16^ are able to demonstrate how regular PROMs data, both short- and long-term, are important in assessing HRQoL in CD patients.

Wright *et al*.^20^ demonstrated consistently lower HRQoL results in women *versus* men, which is in line with previous literature finding similar results in women with CD^33,34^: PCS scores of the SF-36 were found to be significantly lower preoperatively (*P* = 0.029), at 6 months (*P* = 0.017) and at 12 months (*P* < 0.001) following surgery in women compared to men; for the MCS of the SF-36, scores were also significantly lower in women at 6 (*P* < 0.015) and 12 months (*P* = 0.006) following surgery; IBDQ scores were also significantly lower in women at 6 (*P* = 0.007) and 12 months (*P* = 0.006) after surgery. Women reported a greater impact on body image following surgery for IBD than men, which may negatively impact HRQoL^35^.

Abdalla *et al*.^17^ were unable to find differences stratified by sex in assessments of sexual interest and satisfaction. Despite these results, in other studies stomas have been shown to cause adverse effects on a patient’s sexual function and satisfaction, including self-image, embarrassment, dyspareunia, erectile dysfunction, ejaculation and orgasm. It is important to monitor these adverse effects in order to help provide effective, patient-centred care^36,37^. Abdalla *et al*.^17^ justify this difference in results as being caused by the indication for surgery, as most patients in these other stoma-related studies receive surgery due to cancer and malignancy as opposed to CD. In patients with malignancy, more radical, extensive resection may be required, leading to worse physiological outcomes as a result of nerve damage^17^.

Possibly the most unique outcome of the study by Adegbola *et al*.^18^ is the use of the Decisional Regret Scale. Decisional regret is the measure of the patient’s emotion as a result of feelings that better outcomes could have been achieved from opting for a different intervention or having not received the intervention at all and can be an influential factor within decision-making^38^. Negative emotions of decisional regret have been previously associated with worse HRQoL, such as having lower levels of satisfaction as well as being linked to depression and thus are an outcome that should be closely monitored in order to help provide further support for those with these risks of poorer HRQoL^39,40^.

Low levels of patient satisfaction and decisional regret are measures that can be targeted with a push for patient-centred care as well as a shared decision-making process between the clinician and patient, allowing for patients to obtain an increased knowledge surrounding the options available, decreasing levels of uncertainty, providing more realistic expectations of the available outcomes, as well as providing feelings of support^30^. In addition to providing further support, it may also allow for the identification of care pathways lacking in patient involvement within the decision-making process, opening that pathway up for improvement, raising the standard of care in order to minimize patient decisional regret, thus improving overall patient HRQoL.

Koutroumpakis *et al*.^19^ assessed the outcomes of surgery due to conditions potentially secondary to CD. The authors state how the exploration of this pathological pathway is not well established within research and aimed to explore these patients in further detail^19^. The study demonstrated the importance of a universal tool within the greater field of colorectal surgery. The study cohort displayed a broad heterogeneity of participants and if a universal tool was to be utilized across colorectal surgery, the HRQoL of patients with co-morbidities requiring multiple surgeries would be able to be assessed throughout their lifetime. A universal tool would enable the comparison of data before and after the development of a secondary condition without having to use a completely separate new tool and having to translate the information in order for a comparison of outcomes.

With increasing online resources within healthcare due to improvements in technology, catalysed by the coronavirus disease 2019 (COVID-19) pandemic, the advantages of digitally collected PROMs data are becoming more apparent. Patient recruitment and drop-out rates within trials should improve when using digital platforms as less time and effort would be required to recruit patients. They would also be able to access the platform from wherever is convenient, as opposed to being restricted to completing them only when visiting clinics and appointments^41,42^. Furthermore, the results from patients would be directly uploaded to databases without the need for data input from staff, reducing time and costs, streamlining the entire data collection process^42^. This would also negate the issue experienced by Dipasquale *et al*.^14^ where they were required to modify an existing validated tool in order for its administration via the telephone. The IMPACT III questionnaire, a validated assessment of HRQoL in paediatric IBD patients, is a self-administered tool^23^. However, to complete the questionnaire via telephone consultation, 20 of the 35 items in the tools were removed. The modified version of the tool has not been validated and may not produce a full, comprehensive picture of the patient’s HRQoL status, hindering the validity of the results of the study. If the PROM had been made available online, the quality of the tool would not have needed to be compromised.

An online tool with ease of access could lead to a risk of reporting bias, as a result of patients utilizing the tool more frequently when they experience an exacerbation of symptoms. To help reduce this bias, adequate guidance regarding when patients should fill out the tool could prove to be useful. For example, consistent and regular utilization of the tool could be emphasized to patients, and usage patterns could be analysed to identify disproportionate frequency of submissions.

There is no consensus on the best PROM for measuring the HRQoL in patients with CD. The two types of PROMs, generic and disease-specific, targeting different aspects of HRQoL can be complementary if used together. This would produce a holistic view of the patient providing an assessment on how the patient is coping with the specific disease, but also raising red flags for general psychosocial risk. These tools can be collated into a single digital platform and used to gather data from patients with ease, with some suggesting the use of adaptive testing to tailor the relevant questions around the patient using computer algorithms^41^. This digital platform could be expanded from disease-specific to disease-group-specific, not only being used for surgical CD patients but for all IBD patients and even across colorectal surgery, presenting the relevant tools and questionnaires to the specific patient from a single platform (Kontovounisios and Tekkis, unpublished^43^).

The eTHoS trial by Watson *et al*.^44^ is an example of a study utilizing HRQoL as a primary outcome through the use of PROMs to determine quality-adjusted life years within patients undergoing haemorrhoid surgery (stapled haemorrhoidopexy *versus* surgical excision). Watson *et al*.^44^ were able to use the results of the PROMs tools to determine that surgical excision should be considered over stapled haemorrhoidopexy as surgical excision produced significantly better HRQoL outcomes over 24 months post-procedure. Within colorectal surgery, with the impact that CD can have on a patient’s HRQoL, trials should be encouraged to utilize PROMs and patient experience as a primary outcome.

The quality of the systematic review is limited by the types of studies available for analysis. The majority of the articles were of a lower level of scientific evidence, with one of the only three higher-level RCTs that were found being prematurely terminated^15^, making the quality of the data poor. This may also be as a result of this review’s methodology. Searches were limited to only include articles published after 2015. While this might produce data that are within a more consistent timeframe, the absence of older studies could lead to the loss of data as a result.

The tools for measuring HRQoL were also of a limiting nature. Not all of the tools were of the highest quality, with some questionnaires not establishing a minimum clinically important difference for CD, such as PROMIS^17^. The variation in the PROMs used was also limiting in the fact that comparisons of data between studies could not be easily made. This may be due to a lack of consensus on a single universal tool for providing high-quality, valid data in patients with CD.

## Supplementary Material

zrad098_Supplementary_Data

## Data Availability

This systematic review utilized a comprehensive search of relevant literature from online databases. The search strategy, inclusion and exclusion criteria are available upon request from the corresponding author.
